# Localized reconstruction of subunits from electron cryomicroscopy images of macromolecular complexes

**DOI:** 10.1038/ncomms9843

**Published:** 2015-11-04

**Authors:** Serban L. Ilca, Abhay Kotecha, Xiaoyu Sun, Minna M. Poranen, David I. Stuart, Juha T. Huiskonen

**Affiliations:** 1 Division of Structural Biology, Wellcome Trust Centre for Human Genetics, University of Oxford, Roosevelt Drive, Oxford OX3 7BN, UK; 2 Department of Biosciences, University of Helsinki, Viikinkaari 9, 00014 Helsinki, Finland; 3 Diamond Light Source, Harwell Science and Innovation Campus, Didcot OX11 0DE, UK

## Abstract

Electron cryomicroscopy can yield near-atomic resolution structures of highly ordered macromolecular complexes. Often however some subunits bind in a flexible manner, have different symmetry from the rest of the complex, or are present in sub-stoichiometric amounts, limiting the attainable resolution. Here we report a general method for the localized three-dimensional reconstruction of such subunits. After determining the particle orientations, local areas corresponding to the subunits can be extracted and treated as single particles. We demonstrate the method using three examples including a flexible assembly and complexes harbouring subunits with either partial occupancy or mismatched symmetry. Most notably, the method allows accurate fitting of the monomeric RNA-dependent RNA polymerase bound at the threefold axis of symmetry inside a viral capsid, revealing for the first time its exact orientation and interactions with the capsid proteins. Localized reconstruction is expected to provide novel biological insights in a range of challenging biological systems.

## Abstract (web summary)

Electron cryomicroscopy can allow the elucidation of macromolecular structures; however, mismatches in symmetry between different components limit the attainable resolution. Here, the authors set out a computational method for extracting and retaining information from such components.

## Graphical abstract

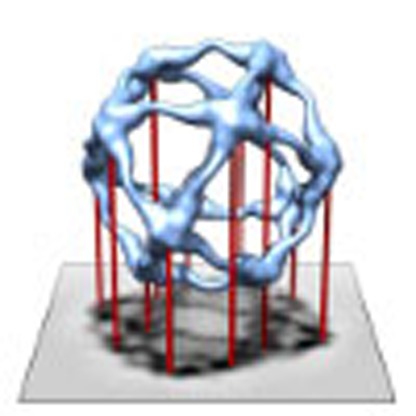

Electron cryomicroscopy (cryo-EM) combined with single particle averaging techniques can provide near-atomic resolution structures of large macromolecular complexes^1^. Quite often these complexes are symmetric allowing further averaging of several asymmetric units, greatly enhancing the signal to noise ratio. However, regions of the structure that deviate from the assumed symmetry are incorrectly averaged and coherent signal is lost. Such deviations from perfect symmetry are common in macromolecular complexes and often a prerequisite for their dynamic functions. Classic examples are the trimeric fibers extending from the five-fold vertices of adenovirus and bacteriophage PRD1 (refs 2, 3). Furthermore, solving structures of viruses in complex with cellular receptors or antibodies has the potential to elucidate virus entry and neutralization mechanisms. However, receptors and antibodies may bind virus capsids in sub-stoichiometric amounts and in a flexible manner^4^,^5^,^6^,^7^.

It is clear that symmetry-mismatched complexes pose a challenge for the structure determination of many macromolecular complexes. For icosahedral viruses, computational approaches have been proposed to tackle one specific class of symmetry mismatches, those occurring at the fivefold vertices of icosahedrally symmetric viruses^8^,^9^,^10^. However, symmetry mismatches are not limited to the fivefold vertices, but can occur at any position in the virus capsid. Furthermore, symmetry mismatches are by no means limited to icosahedrally symmetric assemblies but are also found in many other macromolecular complexes. For example, the GroEL chaperonin that assists substrate proteins in folding is sevenfold symmetric. However, the folding polypeptide, engulfed inside the complex, has no symmetry and may be absent in some of the complexes. Studies of such chaperonin–substrate complexes have exploited image classification to deal with partial occupancy and asymmetric reconstruction to deal with the symmetry mismatch^11^,^12^. Another example is the COPII, a key player in membrane trafficking, which assembles to cages following roughly octahedral symmetry *in vitro*. However, deviations from perfect symmetry have earlier limited the resolution to 30 Å in reconstructions of native COPII cages^13^, and higher resolution (12 Å) has only been achieved after chemical fixation, presumably reducing the flexibility of the cages^14^.

Here, we present a general computational method termed ‘localized reconstruction' for solving structures of symmetry-mismatched parts of macromolecular complexes. Complexes with any symmetry can be analysed and unlike in the earlier ‘vertex reconstruction' method^9^, the structures of interest are not limited to vertices of the complex. The method allows symmetry-mismatched and/or flexible subunits to be treated as isolated single particles separate from the rest of the complex, facilitating their classification, refinement and reconstruction. We apply the method to two published cryo-EM data sets, namely unfixed COPII cages^13^ and the VP4 spikes of triple-layered rotavirus particles^15^, in addition to an unpublished data set of polymerase complexes of double-stranded RNA bacteriophage φ6. In the case of native COPII cages, we show an improvement in resolution from 35 to 14 Å and assess the variation in the subunit binding angles. In the case of rotavirus triple-layered particles, we find, unexpectedly, that not all VP4 sites are occupied. The correct treatment of this partial occupancy allows an improved localized reconstruction of the tip of the VP4 spike. Finally, we present the structure of the φ6 RNA-dependent RNA polymerase at 7.9-Å resolution, demonstrating that the structure can be determined for a symmetry-mismatched, globular 75-kDa enzyme inside a 15-MDa icosahedral viral particle. In conclusion, the presented method opens up a wide range of new possibilities for the structural analysis of symmetry-mismatched complexes by cryo-EM.

## Results

### Method for localized reconstruction

**Figure 1:**
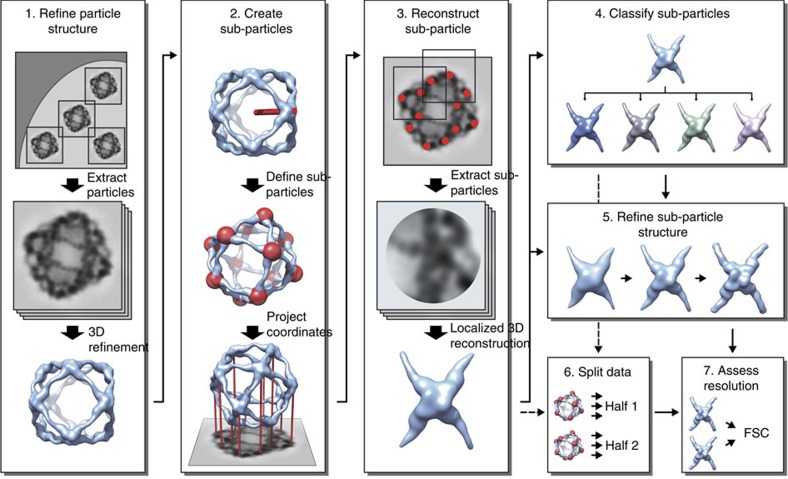
Localized reconstruction of sub-particles from images of macromolecular complexes. Schematic diagram of the workflow for localized reconstruction. First the structure of the macromolecular complex is solved using conventional 3D refinement (1), after which the locations of the sub-structures (red spheres) are calculated based on the particle orientation, a symmetry operator and a vector defining one sub-structure relative to the particle model (red stick; 2). After extracting the sub-particles (red dots) from the particle images, a localized 3D reconstruction is calculated (3). This reconstruction can be used as a starting model for further classification (4) and 3D refinement (5) of sub-particles to improve the structure. Finally two independent sets of data (6) are compared by Fourier shell correlation (FSC) to assess the resolution of the reconstruction (7). See text for a detailed description.

The workflow for localized reconstruction of subunits of macromolecular complexes is shown in Fig. 1. First, conventional three-dimensional (3D) structure refinement, as implemented in many single particle averaging packages, is used to refine the 3D structure of the complex. The next step is to calculate the orientations of the subunits of interest and their positions in the original particle images. We refer to these local areas in the particle images as ‘sub-particles', analogously to sub-tomograms in tomographic volumes^16^. The required inputs for this calculation are: (i) the origin and orientation parameters of each particle, (ii) the symmetry of the macromolecular complex structure, if any, and (iii) a vector defining one subunit structure relative to the structure of the complex (see Methods section). The locations of all sub-particles are calculated and used to extract them from either the original micrographs or from each pre-boxed particle image. In subsequent steps, all sub-particles are treated independently from each another, that is, they are treated as individual single particles in 3D classification, refinement and reconstruction.

As the localized reconstruction method extracts sub-particles from the two-dimensional (2D) projection images, it is unavoidable that some sub-particles overlap the bulk density of the macromolecular complex or each other. Both types of overlaps constitute noise in the images and may thus limit the attainable resolution. Three mutually non-exclusive approaches can be taken to mitigate the effects of overlaps. First, the unwanted, bulk density of the macromolecular complex can be subtracted away in the 2D projection image^10^. Second, side views of the sub-particles can be selected from the areas in the periphery of the particle by giving the maximum allowed angular difference from the plane of the micrograph, and top views of the sub-particles can be excluded. Third, a maximum allowed overlap can be specified by the user, and the sub-particles overlapping more than the maximum allowed overlap can be excluded from further processing.

To demonstrate the application of our localized reconstruction method we interfaced it with Relion^17^, a widely-used package for single particle structure refinement. We apply the method to three data sets (Table 1): native COPII cages^13^, triple-layered rotavirus particles^15^ and bacteriophage φ6 polymerase complexes assembled *in vitro* (previously unpublished).

### Structure of the Sec13/31 vertex in native COPII cages

The COPII cage is a cuboctahedral protein assembly composed of Sec13/31 dimers. Due to its flexible nature, the reconstruction of the unfixed COPII cages has been limited to ∼30 Å resolution^13^. Subsequently, the gradient fixation method^18^ allowed the structure to be resolved at 12-Å resolution^14^. Both of these studies relied on conventional single particle averaging, where octahedral symmetry was imposed.

**Figure 2:**
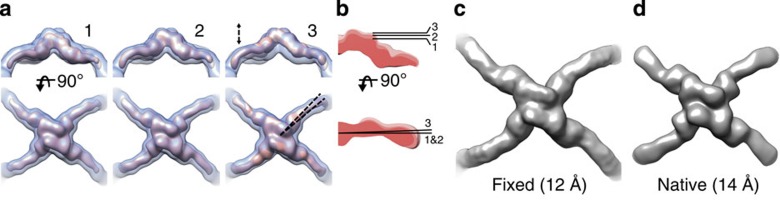
Structural flexibility of Sec13/31 vertices in the native COPII cage. (**a**) Three representative 3D sub-particle class averages (1, 2 and 3) were filtered to 24-Å resolution for comparison and are shown (red) from the side (top row) and from the top (bottom row). The original particle 3D reconstruction (at 35-Å resolution; transparent blue) is shown at a lower threshold to provide a frame of reference. The vertices exhibited two modes of flexibility, in the vertex height and edge angle, as indicated by dashed lines in top and bottom row, respectively. (**b**) A comparison of the three classes (coloured in different shades of red, 1 being the darkest, 2 being medium and 3 being the lightest) is shown as cross-sections of the vertices. The difference between the heights of the vertex in classes 1 and 3 is 15 Å (top row) and the angular difference is two degrees (bottom row). (**c**) The earlier published structure of the fixed COPII cage at 12-Å resolution (EMD-5524)^14^ and (**d**) the localized reconstruction of the native COPII vertex solved in this study to 14-Å resolution are both shown along the twofold axis of symmetry for comparison.

Here, we used the earlier data set of native COPII cages (Table 1, COPII)^13^, to demonstrate the use of localized reconstruction in the case where the particles can be oriented roughly in the 2D projection images, but where flexibility between the subunits limits the attainable resolution in 3D structure refinement. The rationale behind this approach is that when subunits are treated as single particles, it should be possible to achieve higher resolution than for the complex as a whole. First we refined the structure of the whole COPII cage using conventional reconstruction protocols. The resolution of the resulting refined map was somewhat lower (35 Å) than that published before (30 Å)^13^, most likely reflecting the slightly different data processing strategy. Twelve sub-particles, corresponding to the vertices of the octahedral cage, were defined for each particle. Sub-particles were subjected to 3D classification where no shifts or rotations were allowed. The resulting 3D class averages revealed flexibility in the COPII cages (Fig. 2a,b). In one class, the vertices were shifted upwards by 15 Å (Fig. 2a,b, class 3) and rotated by 2° relative to two other classes (Fig. 2a,b, classes 1 and 2). Shifts and rotations of this magnitude most likely explain the limited resolution of the COPII reconstruction by conventional methods^13^. To improve the map by localized reconstruction, sub-particles in the best class were refined allowing small shifts and rotations. The resolution of the resulting map of the native COPII vertex was 14 Å (Fourier shell correlation, FSC=0.143) and showed similar structural details as in the 12-Å resolution reconstruction calculated from fixed COPII cages^14^ (Fig. 2c,d). In conclusion, localized reconstruction first revealed conformational heterogeneity in native COPII cages and then provided a route to improve the resolution.

### The presence and absence of VP4 spikes on rotavirus

**Figure 3:**
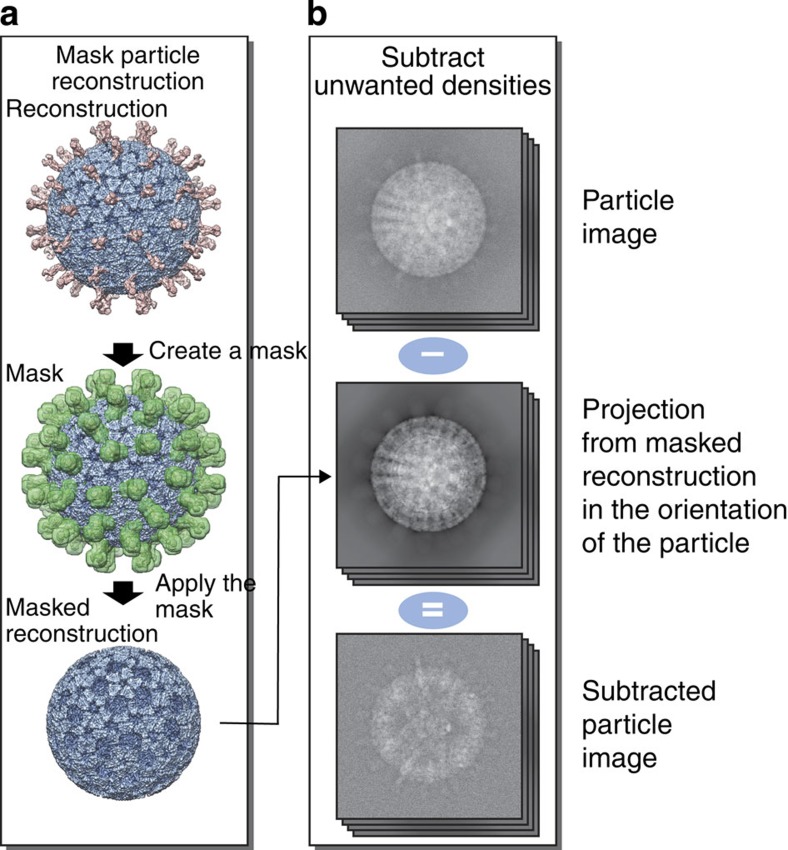
Subtraction of unwanted densities in particle images. Subtraction of densities corresponding to the bulk of the particle is shown in a schematic diagram illustrating a rotavirus triple-layered particle (in blue with external parts of the VP4 spikes in pink). (**a**) First, subunits of interest are removed from the reconstruction of the complex. A mask (green), defining the subunits of interest, is created and then applied on the particle reconstruction to remove densities within the mask. (**b**) The masked reconstruction is projected in the orientations of the particle images (top) to create a stack of masked particle projections (middle). These are then subtracted from the original particle images, to create a new stack of particle images (bottom) that contain density corresponding mainly to the subunits of interest.

Up to 60 copies of the viral protein VP4 form the outermost spike layer of the triple-layered rotavirus particles. The structure of this viral particle has been solved to 4.3 Å resolution, however, the VP4 spikes showed much lower resolution^15^. We hypothesized that this might be due to either partial occupancy or conformational heterogeneity of VP4. We used the data set used in the original work^15^ to study the structure and occupancy of the VP4 spikes using our localized reconstruction method. The locations of 60 VP4 sub-particles were calculated in each of the particles images. To increase the signal for the VP4 spikes, most of the non-VP4 spike components were first subtracted away from the particle images (Fig. 3; see Methods section), and the sub-particles were extracted for further analysis.

**Figure 4:**
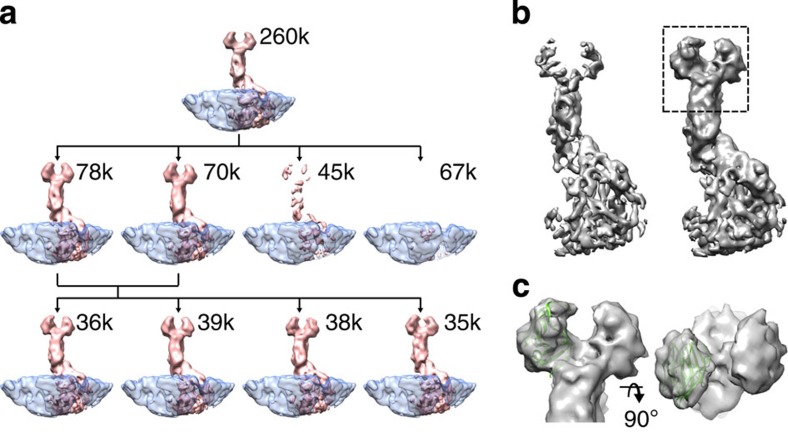
Partial occupancy of the VP4 spike in rotavirus triple-layered particles. (**a**) Three-dimensional classification of VP4 spikes. Class averages (pink) are shown rendered at the same isosurface threshold and resolution (12 Å). The number of sub-particles in each class is indicated. The VP7 density (transparent blue), averaged from all of the sub-particles, is shown as a frame of reference. Sub-particles in the full data set (top row) were first classified into four classes (second row). The first two classes revealed a clear spike density. The sub-particles in these classes were further classified into four classes (bottom row). These classes showed minor differences in the spike conformation. (**b**) Localized reconstructions of the VP4 spike are shown before (∼260,000; left) and after (∼113,000; right) classification and selection of the best sub-particles. Both of the maps have been low-pass filtered to 7.7-Å resolution and rendered at the same isosurface threshold. Most importantly, classification of the sub-particles before reconstruction improved the density for the tip of the spike (rectangle). (**c**) Close-up of the spike tip is shown from the side (left) and the top (right). The structure that forms the two tips of the spike (PDB:4DRR)^19^ has been fitted to the density.

3D classification revealed two classes showing clear VP4 density and, interestingly, two classes where VP4 density was either diminished or absent (Fig. 4a). The VP7 layer was consistent between the classes showing that absence of VP4 in one of the classes was not simply due to preferred orientations of the sub-particles (Supplementary Fig. 1). On the basis of the classification, we estimated the occupancy of VP4 to be 60–70% in this data set. Further classification of the VP4-containing sub-particles revealed only minor differences in the VP4 conformation (Fig. 4a).

3D-localized reconstruction of the sub-particles in the first three classes from the second classification yielded a map showing an improvement in comparison to the original VP4 map calculated before classification (Fig. 4b). Despite the fact that the resolution of the two maps was similar (∼7.7 Å; FSC=0.143), the B-factor for the map calculated after the classification from the best particles was somewhat lower (487 Å^2^) than for the map before classification (523 Å^2^). As a consequence, the map was better resolved and this was true especially for the amino-terminal parts of VP4 forming the two tips of the spike. Fitting of an atomic structure of the spike tip^19^ to the improved map revealed a good fit (Fig. 4c; correlation 0.71), consistent with the previous report^15^.

Despite some success in improving the resolvability of the VP4 spikes, the B-factor of the VP4 spike remained unusually high. This suggests a large amount of conformational heterogeneity that could not be dealt with our current method. The biggest challenge for classification of VP4 sub-particles was the large size of the rotavirus particle and its internal genome. While the contribution of the non-VP4 protein components could accurately be subtracted from the images, this was not possible for the genome component, whose contribution cannot be accurately determined in the 2D projection images as it is not symmetrically ordered in the icosahedrally symmetrized reconstruction.

In conclusion, classification of VP4 sub-particles allowed the occupancy of VP4 in the rotavirus triple-layered particles to be assessed. Surprisingly, we found that a significant fraction of the VP4 sites were unoccupied. Discarding those sub-particles where VP4 was absent before localized reconstruction yielded a better-defined map for the VP4.

### Symmetry-mismatched RNA polymerase of bacteriophage φ6

The bacteriophage φ6 polymerase complex is a dodecahedral assembly formed by 120 copies of the P1 protein, in addition to three minor components: (i) P2, an RNA-dependent RNA polymerase^20^ located at some of the threefold symmetry positions under the P1 shell^21^,^22^, (ii) P4, a hexameric RNA packaging NTPase^23^ located at the fivefold symmetry positions on the P1 shell^24^ and (iii) P7, an assembly co-factor^25^ sharing its binding sites with P2 (ref. 22). *In vivo*, each polymerase complex contains roughly 10 copies of P2 (ref. 26).

**Figure 5:**
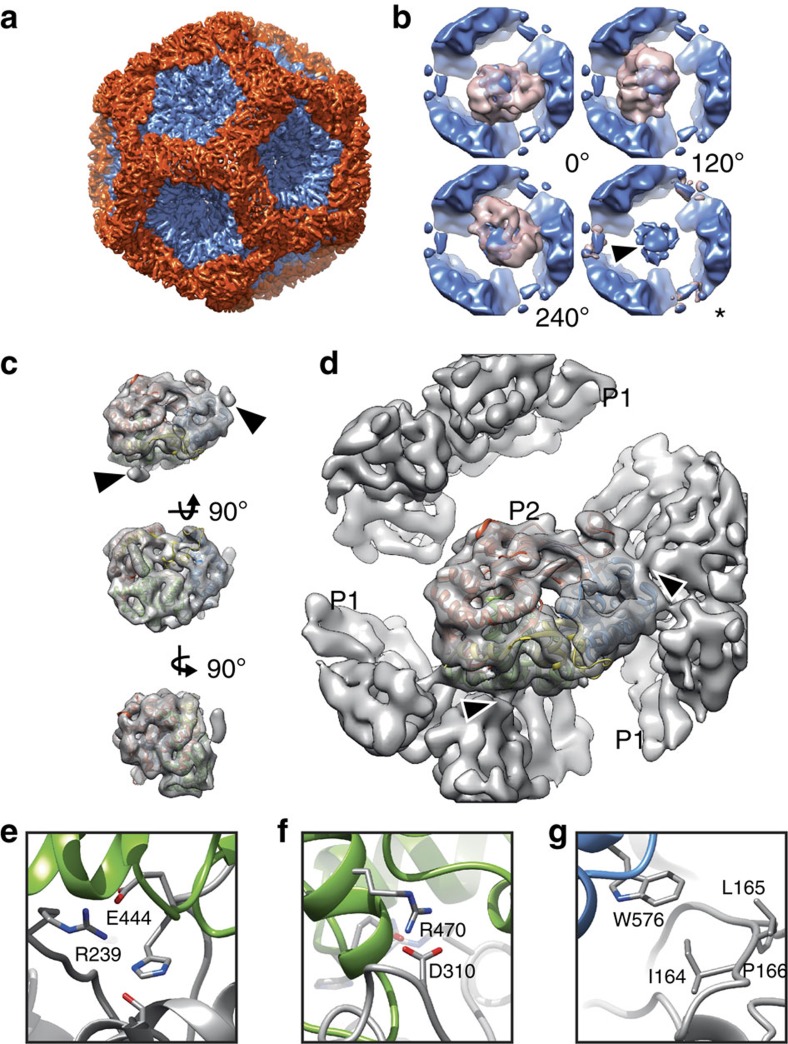
Structure of φ6 P2 polymerase within the polymerase complex. (**a**) Icosahedral reconstruction of the polymerase complex particle at 4.8-Å resolution is shown along the icosahedral threefold axis of symmetry. The P1 monomers around fivefolds are coloured in blue and the P1 monomers around twofolds and threefolds in red. (**b**) Four three-dimensional sub-particle class averages (pink, resolution 12 Å) of the densities under the threefolds and the P1 shell, averaged from all of the sub-particles to provide a frame of reference (blue, resolution 12 Å), are shown. The view is from the inside of the particle. Three classes revealed clear P2 density in the three possible orientations relative to the symmetry axis (0°, 120° and 240°). For another class no P2 density was evident (asterisk). Incorrectly averaged P2 density in the original threefold symmetrized reconstruction is indicated with an arrowhead. (**c**) Localized reconstruction of the P2 density at 7.9-Å resolution with a fitted X-ray structure of P2 (PDB:1HHS) is shown from three different orthogonal orientations. Colouring of the X-ray structure follows the canonical polymerase domain architecture (fingers, red; palm, green; thumb, blue; C terminus, yellow; connecting chains, mauve)^27^. Two small densities, not accounted by the P2 X-ray crystallographic structures, are indicated with arrowheads. (**d**) P2 density, reconstructed together with the P1 shell, is shown in the same orientation as in ***c***. Densities connecting the P2 polymerase to the surrounding P1 shell are marked with arrowheads, and correspond to the densities marked in **c**. (**e**–**g**) Possible contact sites between P2 (the coloured ribbon) and P1 (grey ribbon) are shown and the corresponding amino acid residues labelled.

Here, we used the *in vitro* self-assembly system of φ6 polymerase complexes to make so called ‘P124 particles', consisting of proteins P1, P2 and P4 (ref. 26) with the aim of solving the structure of the P2 polymerase *in situ*. We first solved the structure of the P124 particle using conventional 3D structure refinement assuming icosahedral symmetry (Fig. 5a; Table 1, φ6 P124). The P1 shell was determined at 4.8-Å resolution (FSC=0.143). However, as expected from previous studies^21^, the monomeric P2 polymerase at the threefold symmetry positions under the P1 shell was not resolved (Fig. 5b, arrow head).

Localized reconstruction was used to resolve the density for the symmetry-mismatched P2 polymerase. First, 20 locations of P2 at threefold axis of symmetry were defined for each polymerase complex. P2 sub-particles were extracted from the particle images, where all non-P2 densities had first been subtracted away (see Methods section). The sub-particles were then subjected to 3D classification into five classes. Three classes revealed density corresponding to P2 (Fig. 5b, pink density). As expected, these classes, corresponding to three possible rotations around the threefold symmetry axis (0°, 120° and 240°), contained nearly equal numbers of sub-particles (14,721, 13,912 and 14,583, respectively). The fourth class corresponded to a threefold position, where P2 was absent (Fig. 5b, asterisk; 28,078 sub-particles). Consequently, P2 could be identified in 60% of its putative binding sites in the *in vitro* assembled polymerase complexes resulting in an estimate of 12 copies of P2 per particle on the average. The fifth class (not shown; 16,286 sub-particles) contained most likely misclassified sub-particles or sub-particles that had been extracted from misaligned particles and was not considered in this calculation. In conclusion, 3D classification of the sub-particles was not only sensitive enough to detect the presence or absence of P2 (75 kDa) inside the polymerase complex (15 MDa) but also able to differentiate between the three different possible orientations of P2.

Sub-particles in the three P2-containing classes were used to calculate an asymmetric reconstruction of the P2 polymerase at an estimated resolution of 7.9-Å resolution (FSC=0.143). At this resolution, the complete alpha-helical fold of the protein was clearly resolved (Fig. 5c). Computational rigid-body fitting of the atomic structure of P2 (PDB:1HHS)^27^ into the reconstruction was unambiguous (correlation 0.94) and showed that all of the P2 density could be observed (Fig. 5c).

To resolve possible P1–P2 interactions, we reconstructed P2 in the context of the P1 shell (Fig. 5d). This was achieved by calculating the same reconstruction as above, but this time using the sub-particles extracted from the original particle images, where densities corresponding to P1 were present. This reconstruction revealed the exact orientation of the P2 relative to the P1 shell and two major P1–P2 contact sites (Fig. 5c,d; arrowheads). The first of the two sites contained three putative salt bridges (Fig. 5e,f) and the second site a putative hydrophopic interaction (Fig. 5g). Notably, these interactions differed from those suggested earlier based on P2 rigid-body fitting to threefold averaged density in the icosahedrally symmetric map^21^. Since the side chain conformations for P2 cannot be established at the resolution obtained here, these potential interactions should be checked by site-directed mutagenesis in further studies.

What factors limited the resolution of the P2 reconstruction to 7.9 Å, much lower than achieved for the icosahedrally symmetric P1 shell (4.8 Å)? Firstly, our data set consisted of 4,379 particle images (Table 1). The number of P1 asymmetric units (262,740, due to 60-fold symmetry) was thus much greater than the number of sub-particles with P2 (43,216). Secondly, the B-factor of the P2 reconstruction (515 Å^2^) was a markedly greater than that of the P1 shell reconstruction (200 Å^2^). As the relatively high B-factor could be at least partially due to possible inherent flexibility in the P1–P2 interactions, we sought to improve the P2 structure by refining its orientations. Theoretical calculations suggest that this may be possible even in the case of a protein as small as P2 (75 kDa)^28^, but in practice, to date no structures in this size range (<100 kDa) have been published at comparable resolution (<8 Å) using conventional single particle refinement. Here we benefited from the localized reconstruction approach that defined the approximate positions and orientations of the P2 sub-particles. We thus attempted further refinements of the P2 sub-particles allowing only small changes (±15 degrees) in their orientations and kept their positions fixed. The overall accuracy or rotations as reported by Relion remained low (∼10°) suggesting that the small size of P2 (75 kDa) impeded its orientation refinement, and there was no improvement in the resolution.

In addition to the small size of P2, overlaps between P2 sub-particles constitute noise in the reconstruction leading to elevated B-factors and thus most likely limit the attainable resolution unless the number of sub-particles is increased^29^. To test the effects of overlaps, we discarded the worst overlaps (>40 pixels) in the data set, corresponding to 45% of the total number of sub-particles. We then ran classification identically to the full data set above and three P2-containing classes with 25,054 particles were used to calculate a 3D reconstruction. As expected, removing the overlaps decreased noise in the reconstruction as demonstrated by the decreased B-factor (121 Å^2^). The resolution of this reconstruction was somewhat lower however, 10 Å as estimated by FSC (threshold 0.143; not shown).

Taken together, these results suggest that the relatively small number of sub-particles, overlaps occurring between the particles and the very small size of the polymerase limited the attainable resolution. While in some cases removing overlapping sub-particles may improve the results, here the highest resolution-localized reconstruction of P2 was achieved with the full data set. Despite these limitations, localized reconstruction facilitated determining the P2 structure at 7.9-Å resolution which allowed for the first time an accurate placement of the P2 atomic structure *in situ* and mapping of the P1–P2 interactions.

## Discussion

Many interesting biological structures are not static but undergo conformational changes and exhibit multiple or a continuum of different conformations. Furthermore, complexes often present components in sub-stoichiometric amounts. Understanding this conformational variability is thus key to holistic structural analyses of biological complexes. At the same time, this variability poses a challenge to 3D structure determination by cryo-EM. Gradient fixation has proven to be a useful method in limiting this variability^18^ and facilitated reconstruction of fixed COPII cages to 12-Å resolution^14^. The localized reconstruction method presented here allowed the structure of the COPII vertices to be refined as single particles, circumventing the need for fixation.

In addition to highly symmetrical complexes, subunits that are part of asymmetric complexes are also in principle suitable targets for localized reconstruction. The main requirement is that the macromolecular complex is amenable to single particle averaging and that its orientations can be reliably determined first. In such complexes, areas with lower resolution are often interesting targets for localized reconstruction. These areas can be identified with ResMap, a software that allows quantitative assessment of local resolution^30^. In addition, if the subunit of interest is large enough to be visually identified in the raw images, the ‘dot method', where a computational fiducial marker is added to mark the feature of interest in the raw images, may provide a direct means of identifying the area of interest^4^. Finally, macromolecular complexes that are pleomorphic and thus cannot be treated as single particles can be addressed by tomography combined with sub-tomogram averaging^16^. Indeed, this was the case for COPII assembled on membranes, where sub-tomogram averaging facilitated determining the structure to 40-Å resolution^31^.

To demonstrate how localized reconstruction can be used to address symmetry-mismatched components, we focused on the RNA-dependent RNA polymerase of bacteriophage φ6. Localized reconstruction provided the *in situ* structure of the P2 polymerase to sub-nanometer resolution. The reconstruction was of sufficient quality to allow unambiguous fitting of the known atomic structure of P2 and to reveal possible P1–P2 interactions. Others have previously located the RNA-dependent RNA polymerase under the fivefold symmetry positions in the rotavirus^32^ and under the threefold symmetry positions in bacteriophage φ6 polymerase complex^21^,^22^ using conventional icosahedral reconstruction. Such an approach applies incorrect symmetry to the symmetry-mismatched structures, such as the polymerase in these studies. The application of the localized reconstruction method to the polymerase complexes of φ6 exemplifies how this limitation can be circumvented, allowing the structure of a viral polymerase to be determined *in situ*. This opens up new possibilities for studying RNA replication and transcription in the context of the assembled double-stranded RNA virus capsids.

## Methods

### Calculation of sub-particle refinement parameters

To determine the sub-particle origins, orientations and locations for each particle it is essential to: (i) determine the location of one sub-particle in the reconstruction of a particle calculated in a defined orientation, (ii) determine all the symmetrically related sub-particles in this orientation by using the symmetry of the reconstruction and (iii) determine the parameters for the sub-particles by accounting for the orientation and origin of the particle in each experimental particle image. A computational method to accomplish these tasks was implemented in Python and interfaced with Relion^17^, following standard conventions for electron microscopy image data^33^. The method and documentation are available from http://www.opic.ox.ac.uk/localrec/.

To create parameters for all sub-particles, a vector describing the position of one sub-particle relative to the particle origin is first defined. This can be defined either from the command line or interactively in UCSF Chimera as a Chimera marker file^34^. This vector and symmetry of the particle, if present, are the required inputs for the script *relion_localized_reconstruction.py* together with the particle orientations and origins, read from a Relion-style STAR file. Upon running the script, the sub-particle vector is internally converted to a rotation matrix corresponding to the rotation of a reference vector (that is, [0,0,1]) to the calculated vector. This rotation matrix is multiplied individually by each of the rotation matrices defining the symmetry of the macromolecular complex structure. The resulting matrix products are multiplied by a rotation matrix calculated from the three Euler angles describing the orientations of each particle. The final rotation matrices are used to calculate the three Euler angles defining the orientation of each sub-particle. The parameters of the sub-particles are finally saved in a Relion-style STAR file. The non-integer parts of the sub-particle location coordinates are saved as the origin parameter, to gain sub-pixel accuracy. The sub-particle *Z*-coordinate is used to modify the defocus parameter of the sub-particle, facilitating accurate contrast transfer function (CTF) correction taking into account the thickness of the specimen^35^.

### COPII data processing

We used a previously published set of COPII micrographs (Table 1, COPII) and particle coordinates^13^. CTF parameters were estimated using CTFFIND3 (ref. 36). Particles were extracted from micrographs that showed minimal drift. 3D reconstruction was calculated in Relion^17^,^37^ adhering to the recommended procedures ( www2.mrc-lmb.cam.ac.uk/relion). Octahedral symmetry and a solvent mask with soft edges were imposed during 3D classification and refinement.

The location and orientation of one sub-structure (that is, a Sec13/31 vertex), was defined as a vector starting from the map center and ending in the sub-structure center (at radius of 280 Å) in UCSF Chimera^34^ using the Volume Tracer tool and saving it as a Chimera Marker file. Location coordinates of all sub-particles in all of the particle images were calculated using the defined sub-particle vector and octahedral symmetry matrices in addition to the orientation and origin of each particle. Extracted sub-particles were subjected to 3D classification into four classes in Relion. The final reconstruction was calculated by 3D refinement of the sub-particles from the best class. Resolution was assessed by the Fourier shell correlation (FSC; threshold 0.143) calculated between reconstructions generated from two independent half-sets of the sub-particles^29^.

### Rotavirus triple-layered particle data processing

The rotavirus triple-layered particle data set has been published before (Table 1, rotavirus triple-layered particle)^15^ and was downloaded from http://grigoriefflab.janelia.org/. Processing was carried out essentially as described above for COPII. The refinement parameters were converted from the Frealign (used in the original study) to the Relion convention and an icosahedral reconstruction was calculated in Relion. To assess the resolution the FSC was calculated between reconstructions calculated from two half-sets of particles. The location of one VP4 spike was determined in the reconstruction and coordinates and orientations of all of the sub-particles in the original images were calculated. Differences in the defocus values of the sub-particles at different heights due to the relatively large size of the particle were taken into account in the CTF correction^35^.

To subtract all non-VP4 components in the particle images, fitted atomic coordinates of the VP4 (PDB:3IYU; chains X, Y and Z) were used to generate a binary mask defining one arbitrarily chosen VP4 spike density. Icosahedral symmetry was applied on the mask, and voxels within the symmetrized mask in the reconstruction of the whole particle were set to background level to yield a map defining all non-VP4 components (Fig. 3). This map was used to create a 2D projection for each particle in the matching orientation and defocus, and to further subtract it from the particle image using *relion_project*^17^. VP4 sub-particles were subjected to 3D classification in Relion. To avoid biasing the classification due to the relatively weak signal in the sub-particles, no changes in the orientations or origins were allowed and information only up to 12-Å resolution was included. To limit the effect of high frequencies further, only phase flipping was performed during CTF correction. Final maps of the sub-particles in each class were reconstructed in Relion using full CTF correction and the original sub-particle images. The B-factor was estimated and the inverse B-factor was applied to sharpen the final map for visualization^29^.

### φ6 Polymerase complex self-assembly and purification

For the production of φ6 polymerase complexes with high P2 occupancy the purified φ6 proteins P1, P2 and P4 (refs 20, 25, 38) were mixed in molar ratio 120:120:72 (∼0.2 mg ml^−1^ P1) and incubated in the presence of 6% (w/v) polyethylene glycol 4000 at room temperature for 90 min. To maximize P2 incorporation, P7 was omitted from the self-assembly reaction^25^,^26^. Subsequently, the self-assembled φ6 polymerase complexes (P124 particles) were separated from the unassembled subunits by rate-zonal centrifugation in a linear gradient of 10 to 30% (w/v) sucrose in 20 mM Tris, pH 8.0 (149,800*g*, 80 min, 15 °C). The light-scattering band containing the polymerase complex was collected using a BioComp gradient fractionator (Fredericton, NB, Canada) and the purified fractions were concentrated using an ultra-centrifugal filter device with a 100-kDa cutoff (Amicon; EMD Millipore, Billerica, MA, USA).

An aliquot (3 μl) of purified bacteriophage φ6 polymerase complex (in 20 mM Tris, pH 8.0; 2.4 mg ml^−1^) was applied on a glow-discharged copper grid coated with a film of holey carbon (C-flat; Protochips, Raleigh, NC). Data were acquired using a 300-kV transmission electron microscope (Tecnai F30 ‘Polara'; FEI, Hillsboro, OR) equipped with an energy filter (GIF Quantum LS; Gatan, Pleasanton, CA) with a 20-eV slit width and direct detection camera (K2 Summit; Gatan). Movies (22 frames, each 0.2 s) were collected in electron counting super-resolution mode using a dose rate of 6–8 e^–^ per pixel per s.

### φ6 Polymerase complex data acquisition and processing

Processing was carried out essentially as described above for COPII and rotavirus triple-layered particles. Movies were aligned to account for specimen drift^39^. Movie refinement in addition to particle-based motion correction and radiation damage weighting (‘particle polishing') were performed in Relion following standard protocols^40^. Polished particles were used for calculating the final icosahedrally averaged reconstruction.

All non-P2 components in the particle images were subtracted essentially as for rotavirus triple-layered particles (Fig. 3). First, a spherical mask (diameter 80 Å) defining roughly one incorrectly averaged P2 density under a threefold axis of symmetry in the icosahedrally averaged 3D reconstruction was defined and icosahedral symmetry was applied on the mask. Densities not within the mask were subtracted from the particle images using *relion_project*.

Twenty locations of sub-particles, each centred on a threefold axis of symmetry at a radius of 100 Å, were calculated for each particle. For each sub-particle, one of the three degenerate rotations around the symmetry axis was randomly chosen, so as to not bias the further processing. Sub-particles were classified in five classes using Relion. First, a localized reconstruction of the P2 sub-particle, where no symmetry was applied, was calculated and this was used as an initial template in the 3D classification. As above, a resolution limit of 12 Å was imposed during classification. No alignment was performed and no symmetry was applied. The sub-particles in three of the five classes revealing clear P2 density were combined in one reconstruction (after applying a rotation of 120° or 240° around the symmetry axis as necessary). To assess the resolution and B-factor of P2 in the final reconstruction, a mask defining P2 density was first created in UCSF Chimera by volume segmentation^34^. Finally, the atomic structure of P2 (PDB:1HHS) was fitted into the P2 density in UCSF Chimera by volumetric cross correlation between the P2 density and a map simulated from the atomic structure at 7.9-Å resolution^34^.

To resolve the connections between the P2 and the surrounding P1 shell, the same parameters as used for the reconstruction of P2 alone above were used to calculate a reconstruction from the original sub-particle images (from which no P1 had been subtracted away). This reconstruction was band-pass filtered to 7.9-Å resolution. To identify potential P1–P2 interactions at the atomic level, the atomic structure of the P1 protein (PDB:4K7H)^41^ was fitted in the density map of the capsid as a rigid body using UCSF Chimera. The fitting was improved using real space refinement in COOT^42^ and iterative positional and B-factor refinement in real space using Phenix^43^ and COOT. The initial rigid-body fitting of P2 (PDB:1HHS) was improved by rigid-body refinement in real space using Phenix.

## Additional information

**Accession codes:** Density maps reported in this paper have been deposited in the Electron Microscopy Data Bank under accession numbers EMD-3183 (COPII vertex), EMD-3184 (rotavirus VP4 spike), EMD-3185 (*in vitro* assembled φ6 P124 polymerase complex), EMD-3186 (φ6 P2 polymerase without P1 shell), and EMD-3187 (φ6 P2 polymerase with P1 shell). Atomic coordinates of fitted φ6 proteins have been deposited in the Protein Data Bank under accession codes 5FJ5 (P1 shell of the polymerase complex), 5FJ6 (P2), and 5FJ7 (P2 with P1).

**How to cite this article:** Ilca, S. L. *et al*. Localized reconstruction of subunits from electron cryomicroscopy images of macromolecular complexes. *Nat. Commun.* 6:8843 doi: 10.1038/ncomms9843 (2015).

## Supplementary Material

Supplementary Information Supplementary Figure 1

## Figures and Tables

**Table 1:** Data acquisition and processing statistics.

	**COPII**	**Rotavirus TLP**	**φ6 P124**
*Data acquisition*			
Micrographs	810	110	834
Defocus (μm)*	3.4–6.6	1.2–3.0	1.1–2.6
			
*Structure*			
Size (Å)	600	980	450
Shape	Cuboctahedron	Spherical	Dodecahedral
Symmetry	Octahedral	Icosahedral	Icosahedral
Particles†	5,822 (21,760)	4,339 (4,339)	4,379 (7,635)
Box size (pixels)	288	1,000	480
Pixel size (Å)	2.78	1.23	1.35
Resolution (Å)‡	35	4.1	4.8
B-factor (Å^2^)§	NA	288	200
			
*Sub-structure*	COPII vertex	VP4 spike	P2 polymerase
Size (Å)	300	110	75
Symmetry	C2	C1	C1
Copy number	12	60	20
Sub-particles†	12,663 (69,864)	112,049 (260,340)	43,216 (87,580)
Box size (pixels)	120	80	100
Pixel size (Å)	2.78	2.46	1.35
Resolution (Å)	14	7.7	7.9
B-factor (Å^2^)§	NA	487	515

NA, not applicable; TLP, triple-layered particle.

^*^Positive defocus denotes underfocus.

^†^Number of (sub-)particles used to calculate the reconstruction (the total number in the data set is indicated in parenthesis).

^‡^Resolution (FSC=0.143) indicated for the most ordered part of the density map.

^§^B-factor denotes the overall B-factor approximating also the modulation transfer function of the detector.
