# Supplementary material for: Localized reconstruction of subunits from electron cryomicroscopy images of macromolecular complexes
Source: Nat Commun. 2015 Nov 4;6:8843. doi: 10.1038/ncomms9843 (PMC4667630; doi:10.1038/ncomms9843)
Supplement: Supplementary Information — Supplementary Figure 1 [file ncomms9843-s1.pdf]

Supplementary Figure 1

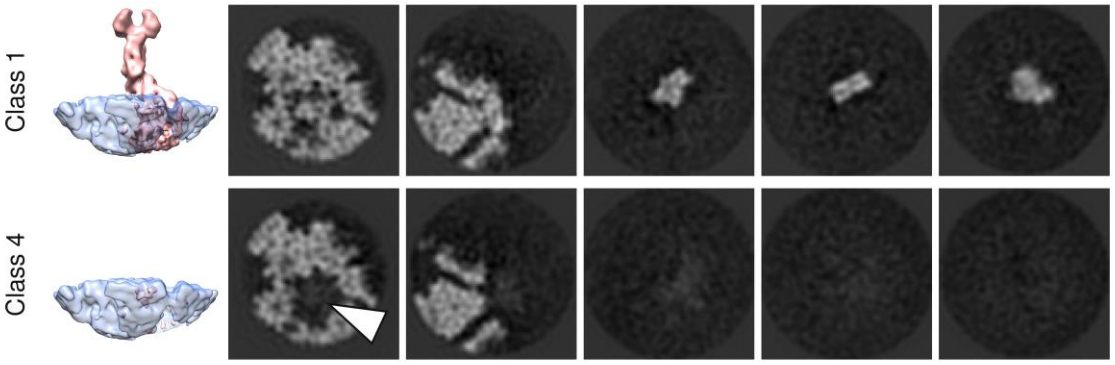

Supplementary Figure 1. Classification of rotavirus VP4 spikes. Two classes (class 1 and class 4) are shown from the classification of rotavirus VP4 spikes. Isosurface representations of the classes are shown on the left. The VLP4 spike (pink) is present in class 1 and absent in class 4. VP7 layer (blue) is shown for frame of reference. Consecutive slices of the density maps (from the level of the VP7 layer on the left towards the tip of the VP4 spike on the right) are shown on the right. A missing VP4 density in the VP7 layer in class 4 is indicated with an arrowhead.
